# Fibular head avulsion fractures accompanying operative treated medial tibial plateau fractures

**DOI:** 10.1007/s00256-019-03191-3

**Published:** 2019-03-05

**Authors:** Tarvo Sillat, Markus Parkkinen, Jan Lindahl, Antti Mustonen, Tatu J. Mäkinen, Rami Madanat, Seppo K. Koskinen

**Affiliations:** 1 Department of Radiology, HUS Medical Imaging Center, Helsinki University Hospital, Helsinki, Finland; 2 Department of Radiology, North Estonia Medical Centre, Tallinn, Estonia; 3 Department of Orthopaedics and Traumatology, Helsinki University Hospital, Helsinki, Finland; 4 Pohjola Sairaala Helsinki, Helsinki, Finland; 5 Department of Clinical Science, Intervention, and Technology, Karolinska Institute, Stockholm, Sweden; 6 Division for Radiology, Department of Clinical Science, Intervention and Technology (CLINTEC), Karolinska Institutet, SE-14186 Stockholm, Sweden

**Keywords:** CT, Trauma, Knee, Fibula, Fracture

## Abstract

**Objective:**

The aims of this work are to determine how frequently medial tibial plateau fractures are accompanied by fibular head avulsion fractures and evaluate the sensitivity of radiographs detecting them, and also to assess if the presence of fibular fracture is correlated with long-term functional outcome and peroneal nerve damage.

**Materials and methods:**

A retrospective chart review of operated patients with medial tibial plateau fractures at level I trauma center during 2002–2008 was performed. From 63 patients imaged preoperatively, 59 had CT and radiographs, three had only CT, and one only radiograph. The presence and fragment size of fibular fracture were retrospectively evaluated. Body mass index (BMI) and functional outcome measurements (the Modified Lysholm knee score and WOMAC) were available for 46 patients.

**Results:**

Fourteen out of 63 patients (22.2%) had fibular fractures. Of the 59 patients with both CT and radiographs, 12 had fibular fractures, and of these, nine were seen with both modalities and three only in CT. Functional scores were available for ten patients with fibular fracture. Patients with fibular fracture seen on radiographs had a significantly higher score on WOMAC function (26 vs. 7; *p* = 0.027). The patients with fibular fractures had also higher BMI (*p* = 0.035). Of the six patients with peroneal nerve damage, 50% had fibular fracture.

**Conclusions:**

In patients with operatively treated medial tibial plateau fracture, the fibular fractures are relatively common. Detecting it is important, as it may be associated with worse functional scores and peroneal nerve paresis. Some fibular fractures may remain undetected on radiographs, hence preoperative CT is recommended.

## Introduction

Tibial plateau fractures are relatively rare with annual incidence approximately10 per 100.000, constituting less than 1–2% of all fractures [1, 2]. Isolated lateral plateau is most commonly involved (55–70%), followed by medial plateau (10–25%) and bicondylar fractures (15%) [3]. Although the isolated medial plateau fractures are relatively rare, they have been regarded to have the worst prognosis [4]. This may be due to the normal biomechanics of the knee, where medial compartment transmits usually more loading than the lateral one [5]. In addition, approximately half of the medial plateau fractures are caused by high-energy trauma [6, 7]. A recent study found that initial articular depression of the plateau measured from preoperative CT scans was a significant predictor of developing post-traumatic OA after operatively treated medial plateau fracture [7].

Medial condyle fractures may have a high prevalence of concomitant soft tissue injuries, e.g., meniscal tears and collateral/cruciate ligament injuries [8]. A common injury mechanism for medial compartment fractures is an axial compression with varus loading that causes distention stresses in lateral structures of the knee. In MRI studies, the lateral collateral ligament (LCL) injuries have been reported even in up to 57% in Schatzker IV fractures [8]. However, LCL injuries requiring operative treatment are less frequent [7]. In addition, especially in younger patients with high-energy trauma, a subluxation or dislocation of the knee with spontaneous relocation may occur. This may increase the risk for concomitant neurovascular injury to the peroneal nerve or popliteal vessels [9] and may necessitate further imaging, e.g., MRI or angiography. However, scientific evidence for these recommendations and also the knowledge about the actual frequency of concomitant neurovascular injuries in isolated medial plateau fractures is still lacking as most of the studies have been conducted on rather small study groups.

Also, the medial plateau fractures are often claimed to be associated with injuries to the posterolateral corner of the knee, such as fibular head avulsion fractures (arcuate sign), although the actual data on the incidence of these are quite limited [10, 11].

The primary objective of this study was to determine how often operatively treated medial tibial plateau fractures were accompanied with fibular head avulsion fractures. The sensitivity of radiographs detecting these fractures was also evaluated with CT as a reference standard. In addition, the presence of fibular fracture was assessed for correlation with long-term functional outcome and permanent peroneal nerve damage.

## Materials and methods

A retrospective chart review of operatively treated patients with tibial plateau fractures at our level I trauma center between 2002 and 2008 was performed and patients with medial tibial plateau fractures were selected and classified according to the AO/OTA [12] classification system, as described previously [7]. In brief, altogether 63 patients were found (mean age 45 years, range 16–86). Of these, 59 patients had both preoperative CT and radiographs, three had only CT images, and one had only radiographs available. These images were retrospectively evaluated independently by two radiologists (a freshly board-certified general radiologist, and a specialist in musculoskeletal (MSK) radiology with more than 15 years of experience in trauma imaging) for the presence of fibular head fractures. In case the fracture was found, the size of the avulsed fragment was measured in three perpendicular directions. This was done to determine if the fractures could be separated in two or more separate groups and if the size would predict the clinical outcome. Equivocal cases were decided by consensus. The patient data were blinded for the radiologists during the evaluation. The mean of the two measurements was used for the analysis.

Functional outcome was assessed as described earlier [7]: valgus and varus laxity were evaluated using manual testing in extension and in 30 degrees of flexion. To assess anterior laxity, the Lachman, anterior drawer, and pivot shift tests were used. Posterior laxity was evaluated using the posterior drawer test. Range of motion was measured using a goniometer. Results were compared to the uninjured contralateral knee. Patients also completed two validated functional outcome measurement tools; the Modified Lysholm knee score and the Western Ontario and McMaster Universities Osteoarthritis index (WOMAC) [13, 14]. Briefly, the Lysholm knee score has eight items of patient-reported measures of knee function, and WOMAC 24 items divided into three subscales: pain, stiffness, and physical function.

Body mass index (BMI) and functional outcome measurement tools (the Modified Lysholm knee score and WOMAC) were available for 46 patients (ten with fibular fractures). The mean follow-up time was 7.6 years (range, 4.7–11.7 years). Mann–Whitney* U* test was used to test the difference in functional outcome between patients with and without fibular head fractures. In addition, association of a permanent injury to peroneal nerve with fibular head fractures was studied with Chi-square test and odds ratio (OR) with 95% confidence intervals (CIs). For statistical analyses, we used a commercial software package SAS/STAT v.9.3 (SAS Institute Inc., Cary, NC, USA). The local ethical committee approved this study.

## Results

**Fig. 1:**
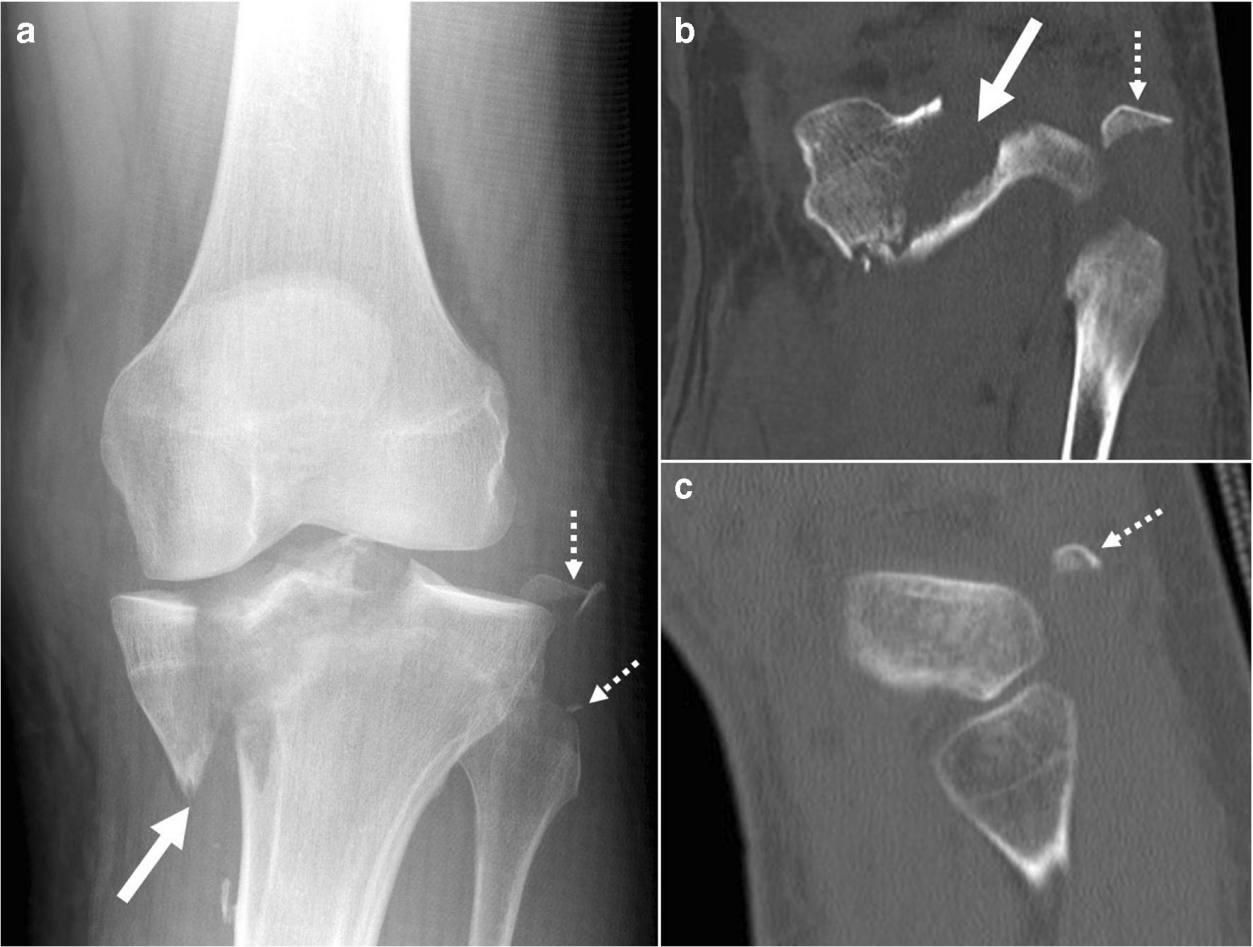
Radiograph (**a**), CT coronal (**b**), and sagittal (**c**) images of 34-year-old male patient who injured his leg by twisting it while playing beach volleyball, show a B3.2 medial plateau fracture (*arrow*) and a fibular head fracture (*dashed arrow*)

Fourteen out of 63 patients (22.2%) had either fibular head or fibular styloid process fractures (Fig. 1). Of the 59 patients that had both CT and radiographs, 12 fractures were found. Of these, nine (75%) were seen with both modalities, and three (25%) only in CT. There was no difference between fractures found by general and specialized MSK radiologist. Of interest is that in original radiology reports of these patients, the presence of fibular fracture was not mentioned in three out of nine available CT reports.

The medial plateau fracture type was according to the AO/OTA classification B1.2 in one case (7%), B3.2 in seven cases (50%), and B3.3 in six cases (43%) (Table 1). In contrast, in patients without fibular head fracture (*n* = 49), the most common fracture type was B3.3 (28 cases; 57%), and the second most common was B3.2 (ten cases; 20%) (Table 1). The difference between different fracture types in patients with and without fibular head fracture was not statistically significant (Chi-square test; *p* = 0.081).

**Table 1:** Fracture type of medial tibial plateau in patients with and without fibular head fracture

Fracture type	Fibular fracture		No fibular fracture	
	*N*	%	*N*	%
B1.2	1	7.1	2	4.1
B1.3	–	–	9	18.4
B3.2	7	50.0	10	20.4
B3.3	6	42.9	28	57.1
Total	14	100	49	100

**Fig. 2:**
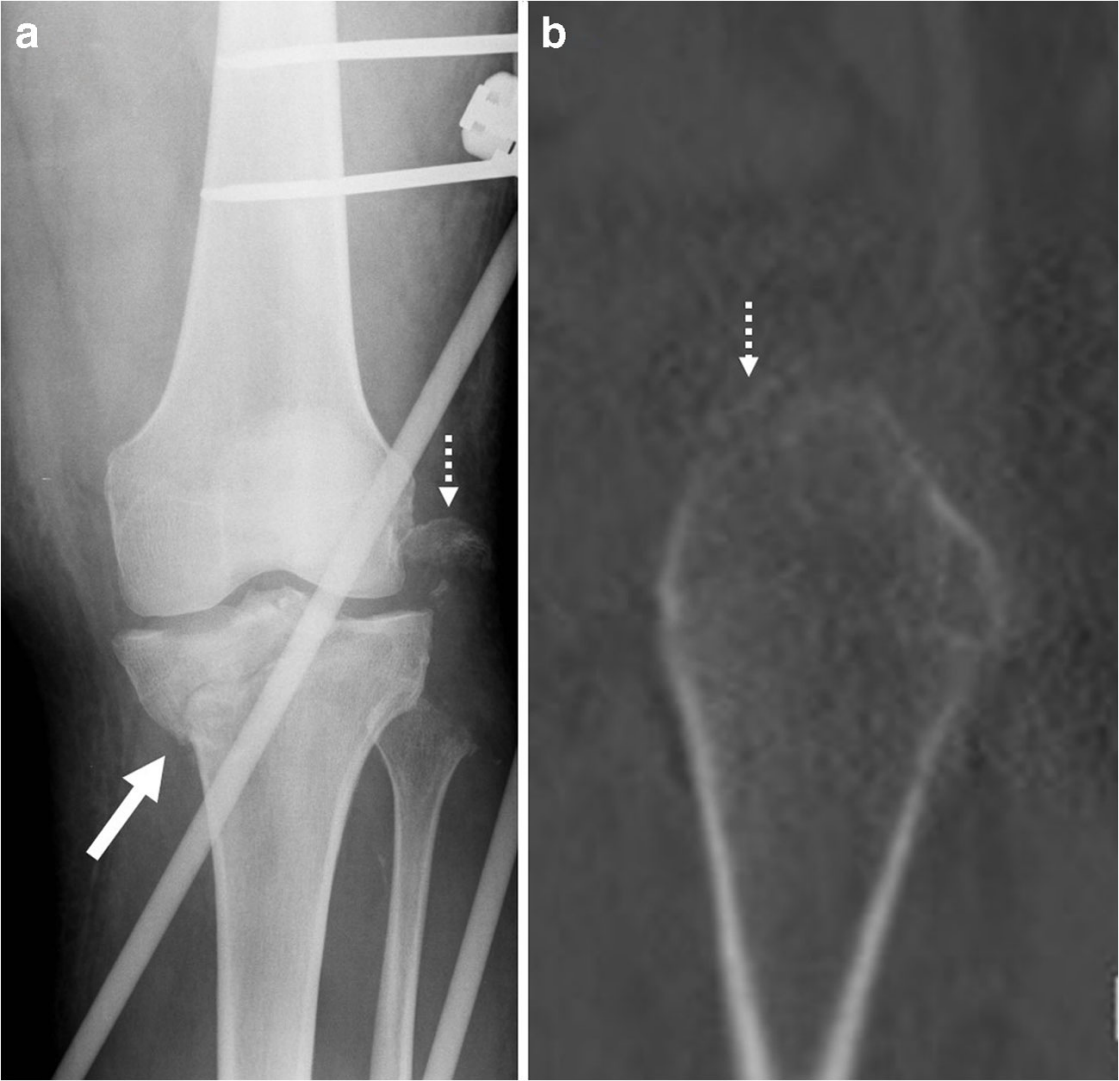
Fibular avulsion fragment sizes ranged in our study from large fragments clearly visible also on radiographs (**a**) to thin curved bone slivers barely seen on CT images (**b**). The radiograph (**a**) belongs to a 46-year-old male patient who fell on the stairs; and the sagittal CT image (**b**) is of a 17-year-old male patient who had a traffic accident while driving a moto-scooter. Both patients had fracture type of B3.3

The size of the fracture fragment (*n* = 18) ranged from 7.0 × 3.0 × 2.0 mm to 28.4 × 14.6 × 18.6 mm (Fig. 2). When the two largest and clearly outlying fragments that involved a significant part of the fibular head, were excluded (Fig. 2a), the rest of the smaller avulsion type of fragments formed based on their fragment size two clearly distinct groups. In first group (nine fragments) the approximate volume measurement of the avulsed fragment remained below 330 mm^3^ (mean = 154 mm^3^, range = 42–330 mm^3^), while in second group (seven fragments) the volumes were above 720 mm^3^ (mea*n* = 962 mm^3^, range = 728–1175 mm^3^).

Of those who had a fibular head fracture, functional scores were available for ten patients. The patients with fibular head fracture seen on radiographs (*n* = 9) had a significantly higher score on WOMAC function (26 vs. 7; *p* = 0.027) (Tables 2 and 3). Also, the patients with fibular head fractures had a significantly higher BMI (*p* = 0.035).

**Table 2:** Age, body mass index (BMI), and functional results of patients with fibular head fractures seen on radiographs

Radiographs	Min	Max	Mean	STD
Fibular fracture (*N* = 9)				
Age	16	73	44.6	27.63
BMI*	20	59	32.3	11.29
Lysholm	51	100	77.3	18.43
Pain	0	60	22	24.12
Stiffness	0	77	24.9	29.72
Function**	0	63	26.1	23.99
No fibular fracture (*N* = 37)				
Age	25	78	45.5	15.75
BMI*	20	38	25.5	17.74
Lysholm	35	100	81.6	15.82
Pain	0	29	7.7	8.33
Stiffness	0	48	8.8	10.41
Function**	0	37	7.1	8.83

*p = 0.035

**p = 0.027

**Table 3:** Age, body mass index (BMI), and functional results of patients with fibular head fractures seen on CT

CT	Min	Max	Mean	STD
Fibular fracture (*N* = 10)				
Age	16	73	43.1	17.25
BMI*	20	59	32.3	11.29
Lysholm	51	100	78.0	17.51
Pain	0	60	19.8	23.78
Stiffness	0	77	22.4	29.12
Function	0	63	23.5	24.08
No fibular fracture (*N* = 36)				
Age	25	78	45.5	15.75
BMI*	20	38	25.5	17.74
Lysholm	35	100	81.6	16.04
Pain	0	29	7.9	8.35
Stiffness	0	48	9.1	10.45
Function	0	37	7.3	8.87

*p = 0.035

There were six patients (6/63; 9.5%) who had a permanent peroneal nerve paresis. Three of them (50%) had a fibular head fracture. The odds ratio for peroneal nerve paresis between patients with fibular head fracture and patients without fibular head fracture was 4.18 (95% CI 0.74–23.59; *p* = 0.085). The fracture volume did not predict the nerve paresis, as the volumes were 42, 995, and 7712 mm^3^. None of the patients had vascular injuries in our series.

## Discussion

In our series of patients with medial tibial plateau fractures, the concomitant bony injury to the fibular head or styloid process was relatively frequent (22%). This probably is reflected by the varus loading mechanism where the medial plateau compression is combined with tension to the posterolateral compartment structures [11, 15], although BMI seems to be another significant factor, as was seen in our study group. Detection of these concomitant injuries is important, as they may be associated with posterolateral rotatory instability of the knee [16]. Therefore, in patients with medial tibial plateau fractures, one should be vigilant for associated proximal fibular head injuries, as they may easily be missed on plain radiographs. In our study, even when these fractures were specifically searched on radiographs, the sensitivity was still only 75%. The reasons for this were suboptimal positioning due to major trauma, overlapping structures, and that the fracture line of the nondisplaced fragment passed obliquely to the X-ray beam in standard projections. In addition, as medial tibial plateau fractures are usually associated with major trauma with certain time-constraints for reporting, it may be that after the more pronounced medial plateau fracture is detected, radiologists tend to overlook much smaller and often non-displaced fibular fractures, or possibly regard them in comparison to tibial fracture not significant enough for mentioning, as was seen in some of the original radiology reports.

When the size of the fibular avulsion fragments was assessed, two size groups were seen. Although further studies are still needed, it is possible that these reflect the two anatomical patterns of arcuate sign that have been described previously: smaller fibular styloid fragments are due to arcuate complex avulsions, while larger fibular head fractures are related to conjoined LCL and biceps tendon avulsion fractures [16].

In our study, a relatively small number of fibular fractures were present, but there was still a significantly higher long-term functional score on WOMAC function in patients with fibular head fracture seen on radiographs. The score was also high in the patient group whose fibular fracture was detected in CT, but due to larger variation the difference was not significant. At the same time, it may also suggest that the more outstanding fractures that were seen in both CT and on radiographs have a higher impact on long-term functional outcome than the small and non-displaced fibular fractures that were seen only on CT.

It is known that medial plateau fractures may be accompanied by peroneal nerve damage [17]. In our study group, the permanent peroneal nerve occurred in 9.5% of the patients, and in patients with fibular fracture, it increased to 21%. In other words, of patients having peroneal nerve damage, 50% had a fibular fracture. Although there are many other factors involved, operatively treated medial plateau fractures with fibular head injury may have a higher risk for peroneal nerve damage. In contrast to previous reports on increased risk for vascular injuries associated with medial plateau fracture, no vascular injuries were noted in our study [18].

In the current study, of the 14 patients with fibular head fracture, only five underwent a lateral ligament reconstruction. Of these five, functional outcome scores were available only for four. This group was too small to evaluate the effect of ligament reconstruction on functional outcome. Also, one of the limitations is the patient inclusion process that was exclusively surgically treated patients, suggesting a selection bias. One may assume that conservative-treated patients with medial tibial plateau fractures revealed a better functional outcome with a lower frequency of fibular head avulsion fractures and lower percentage of permanent peroneal nerve injuries.

In conclusion, in patients with operatively treated medial tibial plateau fracture, the fibular styloid process or fibular head fracture is relatively common. This probably reflects the trauma mechanism where medial compression is combined with tension to the PLC ligaments. Detection of this injury is important, as it may be associated with lower functional scores as well as permanent peroneal nerve paresis. Some proximal fibular fractures may remain undetected on radiographs, hence preoperative CT is recommended.
